# A Psychosocial Exploration of Augmented Reality and Virtual Reality Apps in Cosmetic Procedures

**DOI:** 10.1111/jocd.16612

**Published:** 2024-11-02

**Authors:** Diala Haykal, Hugues Cartier, Frederic Flament

**Affiliations:** ^1^ Centre Laser Palaiseau Palaiseau France; ^2^ Centre Médical Saint Jean Arras France; ^3^ L'Oréal Research and Innovation Clichy France

**Keywords:** augmented reality, cosmetic procedures, decision‐making, patient involvement, patient satisfaction, virtual reality

## Abstract

**Background:**

The integration of augmented reality (AR) and virtual reality (VR) technologies into cosmetic dermatology offers new avenues for enhancing patient engagement, satisfaction, and decision‐making. These immersive tools allow patients to better visualize the outcomes of procedures before treatment, improving communication with physicians and increasing confidence in cosmetic interventions. Despite the promise these technologies hold, there remain concerns regarding their accessibility, technical limitations, ethical challenges, and the potential for algorithmic bias. This commentary aims to explore the psychosocial implications of AR and VR in cosmetic consultations, focusing on their benefits and potential drawbacks.

**Methods:**

This commentary is based on a comprehensive review of literature on AR and VR applications in cosmetic procedures. The focus is on the psychosocial impact of these technologies, highlighting their influence on patient expectations, satisfaction, and decision‐making. Studies that evaluate the integration of AR and VR into cosmetic consultations were analyzed to assess the benefits and challenges associated with these tools.

**Results:**

The analysis revealed that AR and VR significantly enhance patient involvement by allowing real‐time visualization of treatment outcomes, which improves both understanding and satisfaction. Patients reported feeling more informed and confident in their decisions when using these tools during consultations. Furthermore, these technologies allow physicians to offer more personalized and detailed consultations, thus improving communication and alignment of expectations. However, several challenges remain: AR and VR technologies are expensive, their use can lead to discomfort (cybersickness), and algorithmic bias may skew patient perceptions, particularly in underrepresented demographic groups. There are also ethical concerns about data security and transparency of the algorithms used in these systems.

**Conclusion:**

AR and VR hold tremendous potential to revolutionize patient care in cosmetic dermatology by enhancing engagement, improving decision‐making, and increasing overall patient satisfaction. Nonetheless, the full realization of these benefits will require addressing technical and ethical challenges through ongoing research, regulatory oversight, and collaboration between medical professionals and technology developers.

## 1 Introduction

Doctor–patient relationships have undergone a profound transformation in modern health care. Digital platforms provide patients unprecedented access to health information, empowering them as active participants in their care. This marks a significant departure from the traditional doctor‐centered approach, evolving into cooperative treatment planning [1, 2]. Augmented reality (AR) and virtual reality (VR) technologies are at the forefront of this transformation, offering new opportunities to enhance patient engagement, manage expectations, and improve satisfaction and decision‐making in cosmetic procedures. These technologies extend beyond cosmetic dermatology, impacting medical training, patient education, and collaborative decision‐making, with the integration of artificial intelligence (AI) further advancing personalized skincare and fostering patient autonomy [3]. While AR and VR present considerable benefits, it is essential to address the accompanying challenges [2, 4]. Effectively navigating these issues will be crucial to fully leverage the potential of these technologies. This cutting‐edge approach to patient engagement highlights how technology continues to permeate all facets of health care, transform traditional practices, and set new standards for patient involvement and care customization [5]. Ongoing research and thoughtful implementation will be key to ensuring these tools not only revolutionize patient care but also uphold the highest standards of safety and satisfaction.

This study reviews existing literature on the applications of AR and VR in cosmetic dermatological procedures, emphasizing their existing and potential benefits in enhancing care. By leveraging AR and VR, medical professionals can offer more immersive and interactive experiences, which are particularly pertinent in fields like cosmetic dermatology where visual outcomes are critical. While these pros are promising, it is essential to address the accompanying cons to ensure that AR and VR become both useful and safe for clinical use.

## 2 AR and VR in Medical Settings

The first and foremost effect of AR and VR is that these immersive technologies have distinct methods of modifying user perception. However, the mechanisms through which AR and VR create these perceptions are different. AR superimposes digital or virtual content onto the actual physical environment, enabling users to concurrently perceive both the real‐world and virtual components. VR, in contrast, creates fully synthetic, computer‐generated environments that engage users in a three‐dimensional virtual realm. VR separates users from reality, while AR enriches their real‐world experience by providing additional information or interactive elements. AR enhances real‐world interactions using devices like smartphones, tablets, or smart glasses, whereas VR offers complete immersion for activities like gaming and simulations. These technologies have a wide range of uses, along with which the selection between AR and VR is contingent upon the particular use case and the desired degree of immersion and interaction [2]. According to the FDA, AR and VR possess the capacity to revolutionize the field of health care by introducing novel forms of treatments and diagnostics as well as altering the methods and locations of care delivery. Their capacity to provide conventional and innovative content in a highly immersive and realistic manner, remotely, and customized for various clinical scenarios is crucial to their effectiveness in diagnosis and treatment. AR and VR technology can be utilized by physicians, patients, and caregivers to aid in the preparation and execution of specific medical treatments or procedures [6] (Figures 1 and 2). These technologies provide us with a better analysis of body and facial movements and significantly enhance our understanding of physiological and emotional states, especially in younger patients whose key indicators often manifest through facial expressions. A study by Flament, Bazin, and Piot revealed how gravity affects facial signs such as sagging and wrinkling, which helps in medical diagnostics. In the process of perception‐making, these features increase the parity between AR and VR experience of the patient with the real world [7]. Such advancements highlight the transformative potential of AR and VR in healthcare, as supported by research indicating the importance of dynamic and static analysis of facial features in medical diagnostics. Through these technologies, health care can become more accessible, efficient, and tailored to individual needs, fundamentally changing how treatments are administered and experienced. Meanwhile, research by Kurosumi et al. found that dynamic facial movements can accelerate the aging impression in Japanese women, emphasizing the importance of assessing both dynamic and static facial features in medical and cosmetic contexts [8]. When applied to cosmetic dermatology, this information leads to more accurate diagnoses. These technologies also expand tactile and haptic aspects for these applications, crucial for remote examination of skin texture and providing simulated “touch” experiences posttreatment [9]. This integration of tactile feedback in AR and VR enhances understanding of physiological changes or treatment outcomes, offering a more hands‐on examination, even remotely. Such capabilities can revolutionize dermatology and other medical fields, improving diagnostic accuracy and patient engagement without the need for physical presence.

**IMAGE 1:**
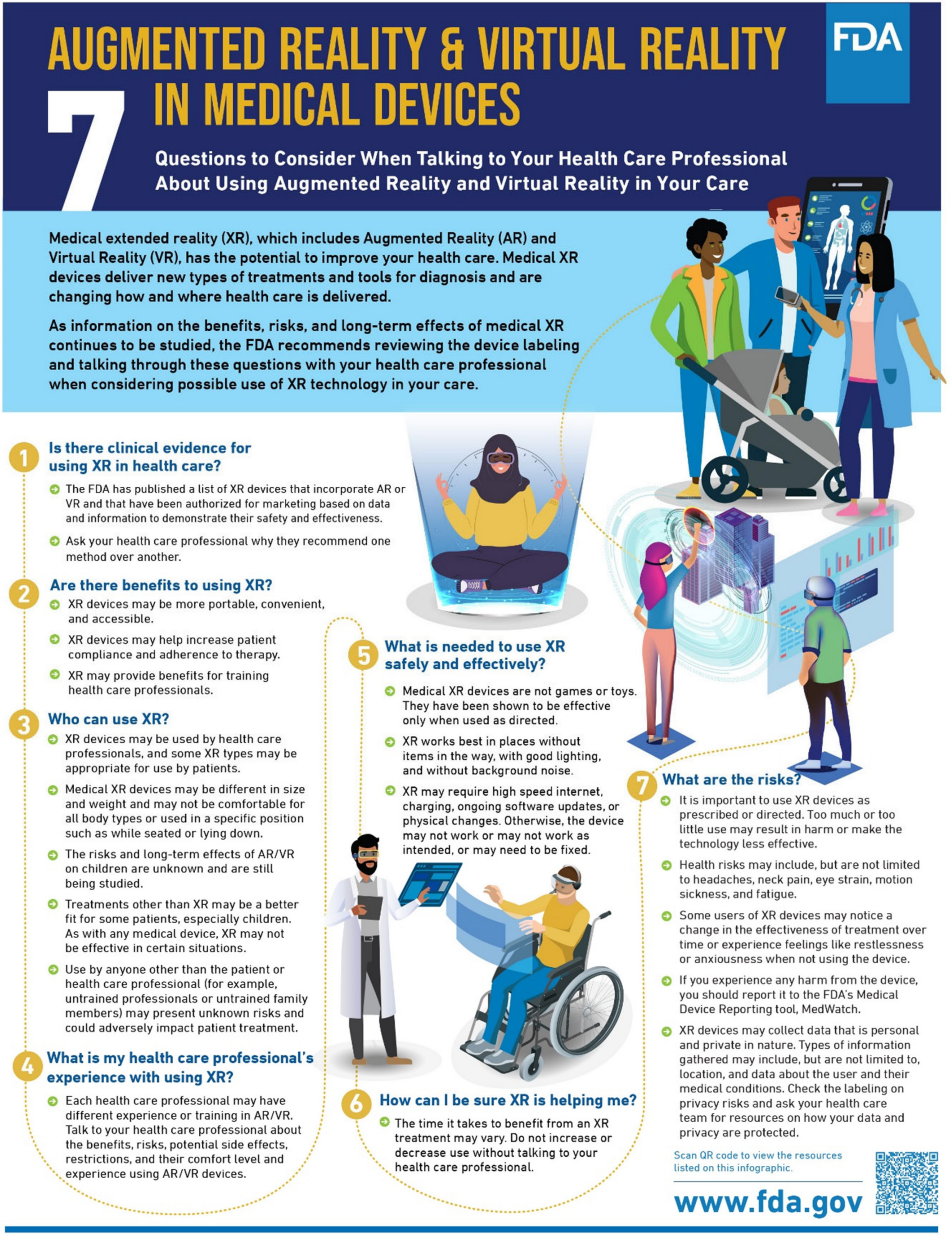
For patients—Questions to consider when talking to your professional about using augmented reality in your care: https://www.fda.gov/medical‐devices/digital‐health‐center‐excellence/augmented‐reality‐and‐virtual‐reality‐medical‐devices‐questions‐consider#Providers.

**IMAGE 2:**
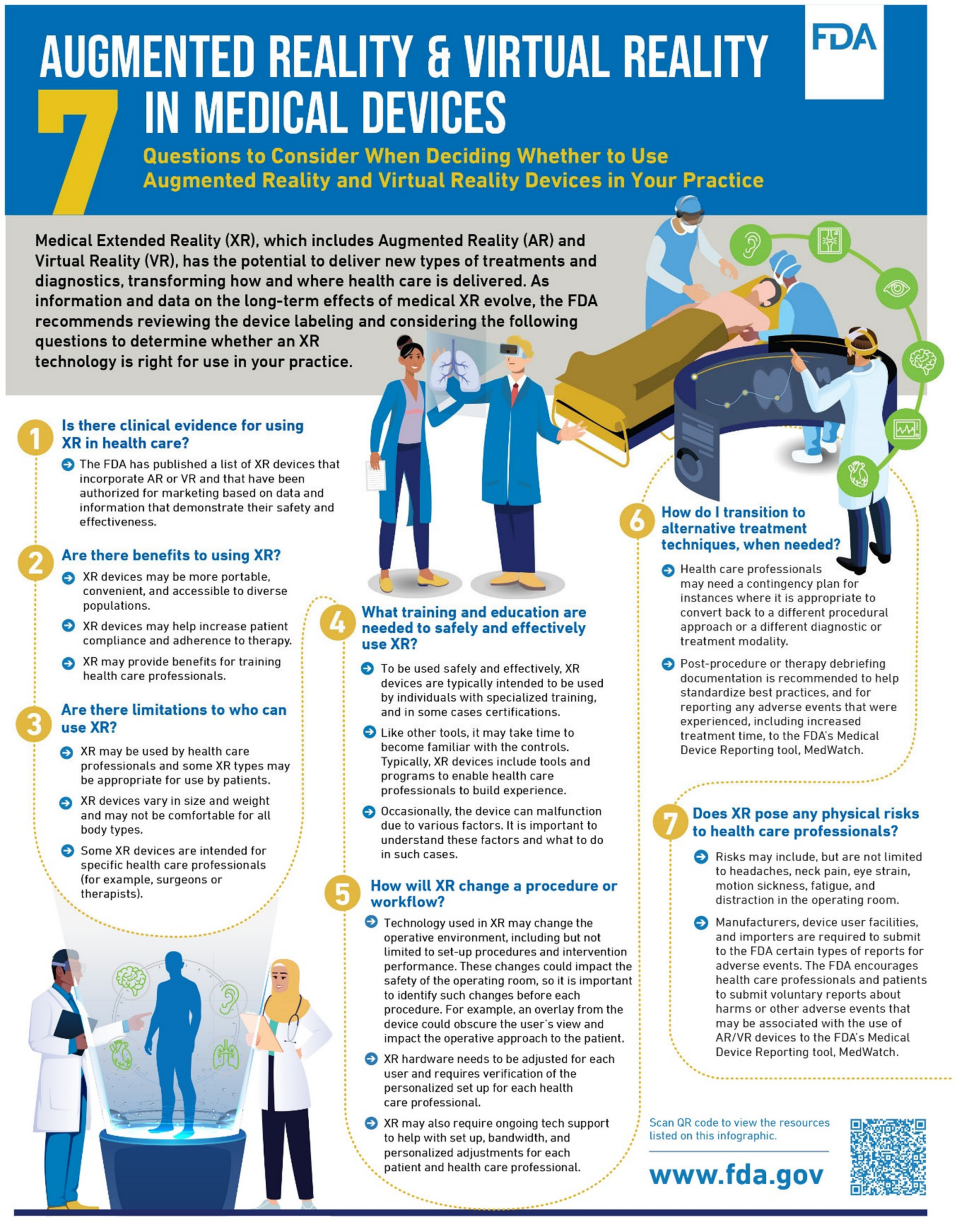
For healthcare providers—Questions to consider when deciding whether to use AR and VR devices in your practice: https://www.fda.gov/medical‐devices/digital‐healthcenter‐excellence/augmented‐reality‐and‐virtual‐reality‐medical‐devices‐questionsconsider#Providers.

Beyond general cosmetic consultations, AR and VR enhance laser‐ and energy‐based medicine, injectables, and body contouring by improving precision, simulating outcomes, enabling practitioner training, and helping patients visualize realistic results, leading to more personalized and accurate treatments [10, 11, 12].

## 3 Patient Involvement for Improved Patient–Physician Relationship

Enhanced patient involvement in medical decision‐making reinforces satisfaction and objective health outcomes while promoting clinical care that is more closely aligned with patient preferences [13]. Recent advancements have focused on patient involvement in areas such as clinical decision‐making, promoting healthy behaviors, and managing chronic conditions. Patient agency in care is rooted in both educational and emotional involvements, with health literacy being crucial [14]. Effective patient education equips individuals to make informed choices, leading to better adherence to treatments and stronger doctor–patient relationships. Ruhnke, Tak, and Meltzer highlight the importance of shared decision‐making, where doctors share information, and patients actively participate, resulting in higher satisfaction and improved health outcomes [15]. Similarly, Krist et al. emphasize the critical role of patient engagement in enhancing overall well‐being and managing both preventive and chronic conditions. Additionally, AR technology enables patients to effectively communicate and educate their families about their medical conditions, further supporting patient involvement [14, 16]. The strong association between patient education and health outcomes emphasizes its crucial role in enabling individuals to actively engage in their own healthcare journey [17]. To transition patients from passive recipients to active participants, decision‐making must be a collaborative process focused on achieving the best outcomes. Healthcare providers can enhance the patient's experience and improve satisfaction by recognizing diverse patient preferences and tailoring their involvement in diagnostic and treatment decisions. In cosmetic dermatology, procedures often trigger a wide range of emotions, from excitement and optimism to anxiety and self‐doubt. Haykal et al. emphasize the importance of understanding these emotional dynamics in enhancing patient care and maintaining ethical practices in the field [18]. Studies show that the factors mentioned above directly impact the success of cosmetic dermatological procedures [19, 20]. The overlap between a patient's emotional and knowledge status and the procedures they undergo requires physicians to take patient education seriously, training them to make informed decisions. Haykal et al. also explore the complex terrain of patient expectations in cosmetic dermatology, uncovering significant discrepancies influenced by cultural and societal differences. These variations highlight the importance of personalized, culturally aware treatment [21]. El‐Haddad, Hegazi, and Hu stress the importance of understanding and quantifying patient expectations to increase satisfaction and provide patient‐centered care [22].

The integration of AR and VR technologies into patient care not only supports better clinical outcomes but also enhances overall well‐being, exemplifying the convergence of technology and patient‐centered care. Recent advances in AR and VR applications in cosmetic dermatology offer accessible and accurate ways for doctors to convey vital information to patients. AR and VR can optimize patient involvement in decision‐making, leading to better outcomes and higher satisfaction.

## 4 Positive Aspects of AR and VR: Influence on Patient Expectations, Satisfaction, and Decision‐Making Processes in Cosmetic Consultations

AR and VR technologies are increasingly being integrated into cosmetic dermatology, fundamentally transforming patient care, expectations, satisfaction, and decision‐making processes. These technologies, often powered by AI, are driving a shift toward more personalized and patient‐centered care by enabling precise visualizations and simulations of cosmetic procedures, thus promoting more informed and empowered patient participation [23]. AR applications have become valuable tools in various subfields of cosmetic dermatology. For example, in laser‐ and energy‐based medicine, AR can be used to map treatment areas with high precision, providing both the patient and the practitioner with a clear understanding of the procedure's scope. This precise mapping is crucial for simulating treatment outcomes and setting realistic patient expectations, thereby enhancing overall satisfaction [10, 11]. Similarly, in injectable procedures, VR offers a simulated environment where practitioners can practice techniques on virtual models, refining their skills before performing on real patients. This not only improves the accuracy and safety of the procedures but also boosts practitioner confidence and patient trust in the treatment process [24]. Additionally, AR applications improve patient education by offering immediate visual representations of skin conditions, raising a more comprehensive perception of diagnoses and treatment alternatives. Virtual try‐ons enable patients to visualize the potential outcomes of cosmetic procedures, contributing to more informed decision‐making [4, 10, 12]. Moreover, incorporating advanced generative adversarial network (GAN) technologies, as demonstrated in research by Despois, Flament, and Perrot further enhances the performance of these applications. GANs can simulate detailed facial reactions and aging processes, potentially extending to tissue or muscle reactions in medical simulations [25]. By improving patients' understanding of their conditions and the procedures they are considering, AR and VR can significantly enhance patient empowerment, leading to better decision‐making and higher satisfaction. In the realm of skincare and beauty, AR and VR are revolutionizing how patients interact with products and treatments. AR applications allow users to conduct real‐time skin analysis and receive customized skincare recommendations, which are often followed by virtual demonstrations for optimal application. These tools enable patients to visualize the effects of skincare products before purchase, fostering transparency, and building trust. Real‐time skin visualization through AR can also demonstrate the immediate impact of products, making the decision‐making process more informed and satisfying [12]. Another benefit is providing a customized virtual skin image analysis which leads to better diagnosis, realistic expectations, and most importantly mentally prepares patients for the procedure [9]. Furthermore, AR‐ and VR‐based teleconsultations have the potential to significantly improve the outcomes of cosmetic procedures. By exposing patients to accurate, detailed information about their care, these technologies help in setting realistic expectations and mentally preparing patients for their procedures. For instance, AR can be used to predict potential complications proactively, allowing for the development of precautionary postoperative care plans [26]. This not only enhances the safety and effectiveness of the procedures but also contributes to better overall patient experiences [11]. The influence of AI in cosmetic dermatology is also noteworthy, as it underpins many of these AR and VR applications. AI‐powered tools are now commonplace, providing 3D facial reconstruction models that forecast clinical results with remarkable accuracy. These advancements are essential in customizing treatments to individual patient needs, further empowering patients by involving them actively in the decision‐making process [27]. Dermatologists are increasingly advised to stay abreast of these technological advancements to continue delivering high‐quality, patient‐centered care [10].

In summary, AR and VR technologies, supported by AI, are transforming cosmetic dermatology by enhancing patient engagement, setting realistic expectations, and improving satisfaction through precise treatment simulations and immersive educational experiences. These technologies empower patients by providing them with accurate information and personalized treatment options, leading to more informed decisions and better outcomes.

## 5 Negative Aspects of AR and VR

To provide a balanced perspective, it is essential to explore the potential drawbacks of AR and VR technologies from both the physician's and the patient's viewpoints. For instance, while AR and VR offer innovative solutions in patient education and procedure visualization, there are concerns about the potential for these technologies to create unrealistic expectations among patients.

### 5.1 Negative Aspects for Patients

From the patient's perspective, one of the primary concerns is the potential for AR and VR to create unrealistic expectations. The highly detailed and idealized simulations presented through these technologies can sometimes lead patients to believe that the postprocedural results will perfectly match the virtual previews. When the actual outcomes differ, it can result in significant disappointment and dissatisfaction, which may exacerbate conditions such as body dysmorphia. Additionally, some patients may experience “cybersickness,” a type of motion sickness triggered by the use of VR headsets, leading to discomfort and limiting the usability of these technologies for certain individuals [28]. Another concern is the issue of data privacy and security. AR and VR systems often require the collection of sensitive personal data, including facial images and detailed treatment plans. If not adequately protected, this data could be vulnerable to breaches, posing a significant risk to patient confidentiality. Moreover, the cost associated with accessing high‐quality AR and VR technologies may create a barrier for some patients, potentially widening the digital divide and limiting access to advanced care options for those from lower socioeconomic backgrounds.

### 5.2 Negative Aspects for Physicians

For physicians, the adoption of AR and VR technologies presents a substantial learning curve. These technologies require significant training and adaptation, which can be time‐consuming and resource‐intensive. Physicians must become proficient not only in the technical use of these tools but also in effectively managing patient expectations and interpreting the data provided by AR and VR systems. There is also a risk that reliance on virtual simulations could reduce hands‐on experience, potentially impacting the development of practical skills that are critical in cosmetic procedures. Furthermore, the high cost of implementing AR and VR technologies can be prohibitive, especially for smaller practices. The need for ongoing updates and the acquisition of new hardware as technology evolves adds to this financial burden, potentially leading to a gap between early adopters and those who cannot afford continuous upgrades. Additionally, there are ethical concerns related to the biases that may be embedded in the algorithms used by AR and VR systems. These biases could lead to unequal treatment outcomes, particularly if the technology is not calibrated to account for diverse patient populations with varying skin tones, facial structures, and other characteristics. The immersive nature of AR and VR might lead some patients to expect outcomes that are not feasible, which can result in patient dissatisfaction, which in turn might gravely lower successful rates of our clinics [25]. These factors must be weighed against the benefits when considering the implementation of AR and VR in clinical settings [4, 29]. Moreover, there is a critical need for regulatory oversight to ensure these technologies are used responsibly, preventing potential misuse and safeguarding both patient expectations and clinical outcomes.

## 6 AR and VR Influence on Physicians' Training and Skills

The incorporation of VR technologies has the capacity to fundamentally transform the training of clinical and technical abilities. VR provides healthcare professionals with a dynamic platform to enhance their skills in a safe and realistic simulated environment. VR training can serve as a valuable adjunct to conventional approaches, enabling practitioners to rehearse and achieve mastery in complex procedures where accuracy and proficiency are of utmost importance. This not only hones their skills but also enhances patient safety and outcomes, ensuring that medical professionals are adequately prepared and skilled in their practice [30, 31, 32]. Augmented and mixed reality technologies are revolutionary tools in the field of surgery, greatly improving outcomes by optimizing navigational procedures. These technologies provide surgeons with an immersive and detailed perspective of the surgical area, enabling real‐time, three‐dimensional visualizations that improve spatial awareness. AR enhances the process of making navigational decisions by superimposing digital information onto the physical environment. This enables surgeons to quickly identify important structures and navigate intricate anatomical landscapes with enhanced accuracy [11, 33]. This integration could provide an even more nuanced and effective training tool, closely mimicking real‐life scenarios for medical professionals.

## 7 Discussion

The integration of AR and VR technologies in cosmetic dermatology offers transformative potential by enabling patients to visualize treatment outcomes more accurately, thereby improving engagement, decision‐making, and overall satisfaction. These technologies allow patients to see detailed simulations of procedures, which can help align their expectations with realistic outcomes. However, these benefits are accompanied by significant challenges [34]. One of the primary concerns is the risk of creating unrealistic expectations, as the idealized outcomes shown in AR and VR simulations may not always be achievable, leading to potential dissatisfaction. Additionally, some patients may experience discomfort, such as “cybersickness,” when using VR, which can limit the accessibility of these tools [28]. Data privacy and security are also critical issues, as AR and VR technologies often involve the collection of sensitive personal information, including facial images and treatment plans, which must be protected against breaches. For physicians, AR and VR provide innovative tools that can enhance the precision of procedures and improve patient communication. However, the high cost of updating AR‐ and VR‐based systems, the risk of data bias and the possible demise of essential hands‐on skills are some of the most important downsides accompanying the use of these systems. To fully realize the potential of AR and VR in cosmetic dermatology, it is essential to approach their integration with careful consideration of these challenges. This involves not only ensuring that practitioners are adequately trained and that patient expectations are managed realistically but also implementing robust measures and global guidelines to protect data privacy and address algorithmic biases [35, 36]. By thoughtfully balancing the innovative capabilities of AR and VR with these critical considerations, these technologies can be effectively integrated into clinical practice, ultimately enhancing patient care and advancing the field of cosmetic dermatology [3, 5, 37, 38, 39, 40].

## 8 Conclusion

Ultimately, the evolving healthcare environment, characterized by patient empowerment, technological integration, and individualized care, is fundamentally reshaping the doctor–patient dynamic. AR and VR technologies have already begun transforming cosmetic procedures by enhancing patient engagement, managing expectations, and improving satisfaction and decision‐making. Beyond cosmetic dermatology, these immersive technologies have significant potential in medical training, patient education, and collaborative decision‐making, further amplified by AI integration, which fosters personalized cosmetic procedures, skincare, and patient autonomy. However, it is crucial to recognize the challenges associated with AR and VR, such as potentially unrealistic patient expectations, the learning curve for practitioners, high costs, and ethical concerns, including data privacy and algorithmic biases. These issues must be carefully managed to fully realize the benefits of these technologies. As AR and VR continue to evolve, they offer exciting new horizons in clinical and technical skills training, providing secure, lifelike environments for practitioners, and more personalized experiences for patients. The ongoing exploration and research into AR and VR applications will be essential to ensuring their ethical and effective integration into medical practice, ultimately leading to a better‐informed and actively engaged society.

In summary, AR and VR technologies are poised to revolutionize cosmetic dermatology and broader health care, but their successful adoption will depend on balancing innovation with careful consideration of the associated challenges, ensuring these tools enhance patient care and satisfaction without compromising ethical standards.

## Author Contributions

D.H. wrote the manuscript, and H.C. and F.F. reviewed it.

## Ethics Statement

The authors have nothing to report.

## Consent

The authors have nothing to report.

## Conflicts of Interest

The authors declare no conflicts of interest.

## Data Availability

The data that support the findings of this study are available upon references' part.
